# Elongase Reactions as Control Points in Long-Chain Polyunsaturated Fatty Acid Synthesis

**DOI:** 10.1371/journal.pone.0029662

**Published:** 2011-12-22

**Authors:** Melissa K. Gregory, Robert A. Gibson, Rebecca J. Cook-Johnson, Leslie G. Cleland, Michael J. James

**Affiliations:** 1 Rheumatology Unit, Royal Adelaide Hospital, Adelaide, Australia; 2 Food Science, School of Agriculture, Food and Wine, University of Adelaide, Adelaide, Australia; Governmental Technical Research Centre of Finland, Finland

## Abstract

**Background:**

Δ6-Desaturase (Fads2) is widely regarded as rate-limiting in the conversion of dietary α-linolenic acid (18:3n-3; ALA) to the long-chain omega-3 polyunsaturated fatty acid docosahexaenoic acid (22:6n-3; DHA). However, increasing dietary ALA or the direct Fads2 product, stearidonic acid (18:4n-3; SDA), increases tissue levels of eicosapentaenoic acid (20:5n-3; EPA) and docosapentaenoic acid (22:5n-3; DPA), but not DHA. These observations suggest that one or more control points must exist beyond ALA metabolism by Fads2. One possible control point is a second reaction involving Fads2 itself, since this enzyme catalyses desaturation of 24:5n-3 to 24:6n-3, as well as ALA to SDA. However, metabolism of EPA and DPA both require elongation reactions. This study examined the activities of two elongase enzymes as well as the second reaction of Fads2 in order to concentrate on the metabolism of EPA to DHA.

**Methodology/Principal Findings:**

The substrate selectivities, competitive substrate interactions and dose response curves of the rat elongases, Elovl2 and Elovl5 were determined after expression of the enzymes in yeast. The competitive substrate interactions for rat Fads2 were also examined. Rat Elovl2 was active with C_20_ and C_22_ polyunsaturated fatty acids and this single enzyme catalysed the sequential elongation reactions of EPA→DPA→24:5n-3. The second reaction DPA→24:5n-3 appeared to be saturated at substrate concentrations not saturating for the first reaction EPA→DPA. ALA dose-dependently inhibited Fads2 conversion of 24:5n-3 to 24:6n-3.

**Conclusions:**

The competition between ALA and 24:5n-3 for Fads2 may explain the decrease in DHA levels observed after certain intakes of dietary ALA have been exceeded. In addition, the apparent saturation of the second Elovl2 reaction, DPA→24:5n-3, provides further explanations for the accumulation of DPA when ALA, SDA or EPA is provided in the diet. This study suggests that Elovl2 will be critical in understanding if DHA synthesis can be increased by dietary means.

## Introduction

Metabolism of the vegetable-derived omega-3 (n-3) fatty acid, α-linolenic acid (18:3n-3; ALA), to the long-chain n-3 polyunsaturated fatty acids (PUFA), eicosapentaenoic acid (20:5n-3; EPA) and docosahexaenoic acid (22:6n-3; DHA), requires progressive desaturation and elongation. The location of regulatory steps in the pathway has largely been inferred from the intermediate fatty acids that accumulate in tissues following increased exposure to ALA. With increasing abundance of ALA or stearidonic acid (18:4n-3; SDA), which is the direct Δ6-desaturase (Fads2) product of ALA desaturation, the next metabolite to accumulate is EPA [1], [2], [3]. Although it is commonly reported that Fads2 is rate-limiting for the metabolism of ALA to DHA, the accumulation of EPA and not DHA suggests other control points need to be considered [1], [2], [3]. As EPA increases, the next metabolite to accumulate is the elongation product docosapentaenoic acid (22:5n-3, DPA) [3]. The accumulation of EPA and DPA merit a systematic examination of the elongase enzymes involved in their metabolism. Human and rat elongase-5 (Elovl5) elongate C_18_ and C_20_ PUFA [4], [5] and human and mouse elongase-2 (Elovl2) are active with C_20_ and C_22_ PUFA [6]. However, the putative substrate preferences for these enzymes have been inferred from separate studies, each using single concentrations of substrate. Accordingly, these studies provide little insight into the reasons for EPA and/or DPA accumulation when upstream precursor fatty acids are abundant.

Beyond DPA formation, Fads2 may have an additional influence on the pathway. In addition to metabolising ALA, it also metabolises 24:5n-3, a progenitor of DHA. Since Fads2 is used by two substrates in the pathway from ALA to DHA synthesis, there is intrinsic potential for competitive substrate inhibition. Substrate inhibition has been suggested as an explanation for increasing EPA and DPA, but decreasing DHA when dietary ALA is abundant [7], [8]. However, direct competition between ALA and 24:5n-3 with regard to Fads2 has not been reported.

We report herein comparative studies of the rat elongases with regard to expression, substrate selectivities, competitive substrate interactions, and dose response curves. We also examined the possible competitive substrate interactions for rat Fads2. The enzyme activity studies used a yeast heterologous expression system.

## Results

### Elongases

#### Sequence analysis of *Elovl2* and *Elovl5*

Amplification of the 840 bp *Elovl2* ORF and subsequent alignment with the rat *Elovl2* sequence in GenBank (NM_001109118) revealed the two sequences were identical. Amplification of the 900 bp *Elovl5* ORF and subsequent alignment with the rat *Elovl5* sequence in GenBank (NM_134382) revealed the two sequences had four nucleotide differences. The *Elovl5* sequence has been deposited in GenBank as accession number HQ404314. The predicted Elovl2 and Elovl5 proteins of 279 and 299 amino acids, respectively, included all of the characteristic features of a microsomal fatty acyl elongase and had 56% identity. However, due to the nucleotide changes in the *Elovl5* sequence, the predicted Elovl5 protein sequence included four residue differences from the rat Elovl5 sequence in GenBank (NP_599209). The Elovl2 and Elovl5 proteins are predicted to contain five and six transmembrane regions, respectively (SOSUI software, http://bp.nuap.nagoya-u.ac.jp/sosui/).

#### Comparison of Elovl2 and Elovl5 substrate specificities

**Figure 1:**
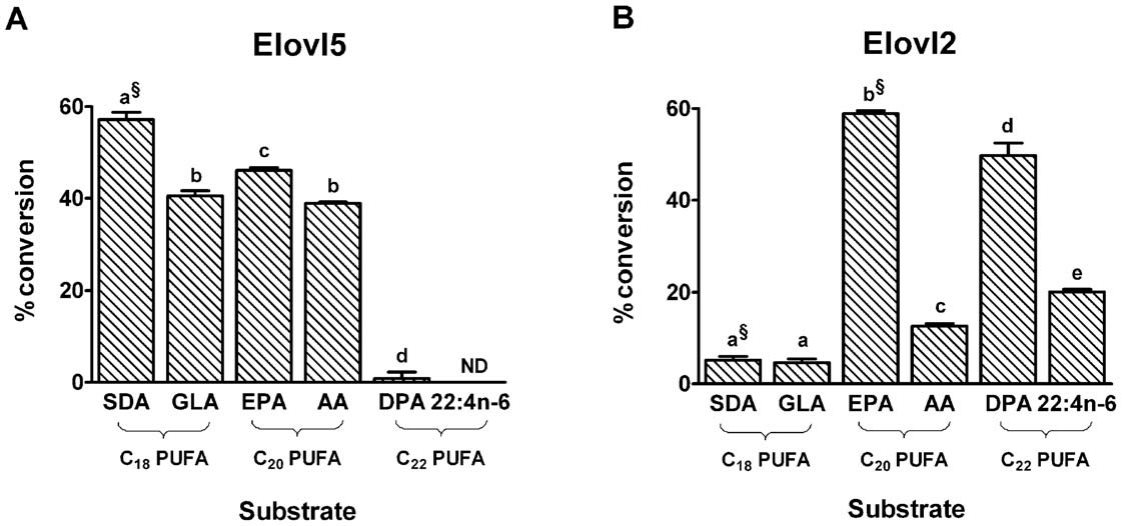
Fgure 1. Comparison of the rat Elovl5 and Elovl2 substrate specificities. Recombinant *S. cerevisiae* expressing Elovl5 (A) or Elovl2 (B) were grown in the presence of 100 µM of various C_18_, C_20_ and C_22_ PUFA substrates. Fatty acids were extracted from the recombinant *S. cerevisiae* and the amount of each fatty acid was expressed as a percentage of the total amount of all fatty acids. The proportion of substrate fatty acid converted to longer chain fatty acid product(s) was calculated as [product(s)/(product(s) + substrate)]×100. The product was 2 carbons longer than the substrate. ^§^ denotes that the conversion includes the 4 carbon elongation product. The results are the means ± S.D. of triplicate incubations. Values with different symbols are significantly different from each other. ND, not detected.

Recombinant *S. cerevisiae* cells expressing Elovl2 or Elovl5 were cultured in the presence of 100 µM of various C_18-22_ PUFA to determine substrate specificities of the elongases. Elovl5 demonstrated elongase activity with C_18_ and C_20_, but not C_22_ PUFA (Fig. 1a). By contrast, Elovl2 demonstrated little activity with C_18_ PUFA, but was active with C_20_ and C_22_ PUFA substrates (Fig. 1b). The Elovl2 activity was 4.5- and 2.5-fold higher with the n-3 substrates EPA and DPA, respectively, compared with their n-6 homologs, arachidonic acid and 22:4n-6.

#### Competition between n-3 substrates for Elovl2 and Elovl5

**Figure 2:**
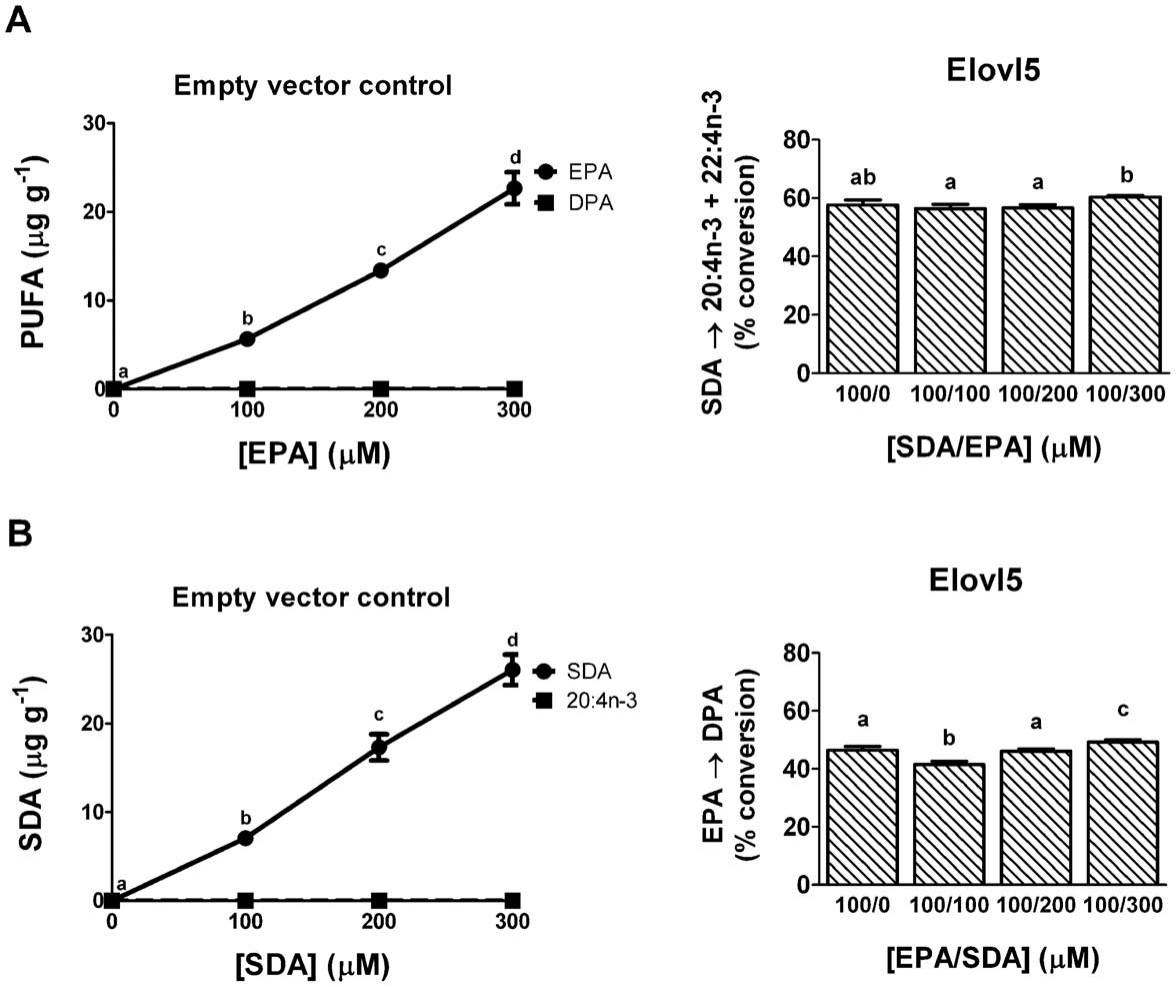
Competition between n-3 PUFA substrates for elongation by Elovl5. Recombinant *S. cerevisiae* expressing Elovl5 were grown in the presence of 100 µM of SDA and 0–300 µM EPA (A) or 100 µM of EPA and 0–300 µM SDA (B). Recombinant *S. cerevisiae* containing the empty pYES2 vector were grown in the presence of 0–300 µM EPA or SDA. Fatty acids were extracted from the recombinant *S. cerevisiae* and the amount of each fatty acid was quantified in µg g^−1^ of *S. cerevisiae* or expressed as a percentage of the total amount of all fatty acids. The proportion of substrate fatty acid converted to longer chain fatty acid product(s) was calculated as [product(s)/(product(s) + substrate)]×100. The conversion of SDA includes the 4 carbon elongation product 22:4n-3. The results are the means ± S.D. of triplicate incubations. Values with different symbols are significantly different from each other.

Since both SDA and its downstream product, EPA, are substrates for Elovl5, there is the possibility of competition for enzyme activity. However, over the concentration range investigated there was no competitive inhibition of EPA elongation by SDA, or SDA elongation by EPA (Fig. 2). The elongation products of SDA and EPA were not detected in the empty vector dose response experiments (Fig. 2). Elovl2 also has two n-3 substrates, EPA and DPA. However, attempts to examine possible competition between EPA and DPA were problematic due to Elovl2 performing sequential reactions with EPA such that DPA is both product and substrate. The sequential reactions with Elovl2 were examined further.

#### Examination of product/substrate relationships for Elovl2 and Elovl5

**Figure 3:**
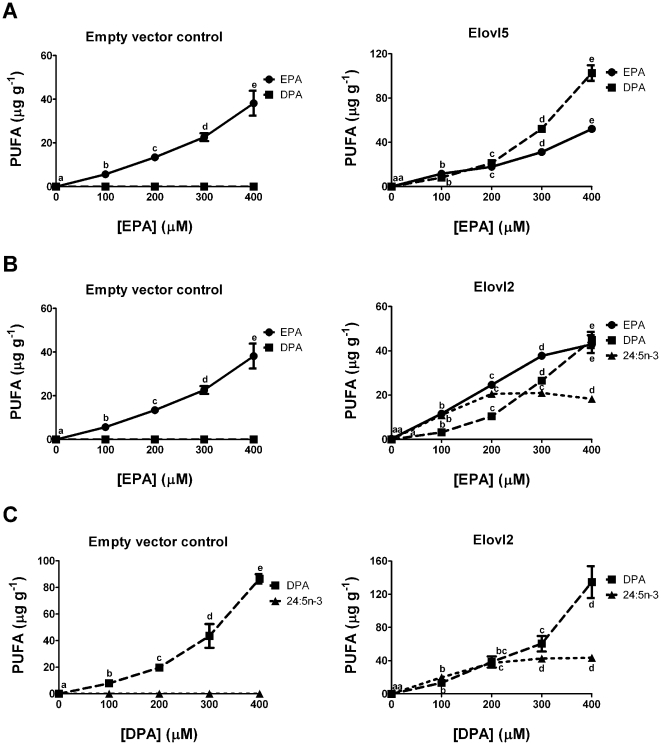
Examination of product/substrate relationships for elongation by Elovl5 and Elovl2. Recombinant *S. cerevisiae* expressing empty pYES2 vector or Elovl5 were grown in the presence of EPA (A) or recombinant *S. cerevisiae* expressing empty pYES2 vector or Elovl2 were grown in the presence of EPA (B) or DPA (C). Fatty acids were extracted from the recombinant *S. cerevisiae* and the amount of each fatty acid was quantified in µg g^−1^ of *S. cerevisiae*. The results are the means ± S.D. of triplicate incubations. Values with different symbols are significantly different from each other.

The initial results indicate that EPA at 100 µM can be used efficiently as a substrate by both Elovl2 and Elovl5. For further comparisons, EPA dose response curves for the two enzymes were made. For Elovl5 there was a proportional increase in DPA synthesis with increasing EPA concentration (Fig. 3a). For Elovl2 there was also a proportional increase in the product of the first reaction, DPA, but not the product of the second reaction, 24:5n-3 (Fig. 3b). This may suggest that the second reaction of endogenously generated DPA conversion to 24:5n-3 is saturated at concentrations of EPA that are not saturating for the first reaction, EPA conversion to DPA. When DPA was used as an exogenous substrate, apparent saturation of conversion to 24:5n-3 was also observed (Fig. 3c). The elongation products of EPA and DPA were not detected in the empty vector dose response experiments (Fig. 3).

#### Expression of *Elovl2* and *Elovl5* in rat liver and heart

**Figure 4:**
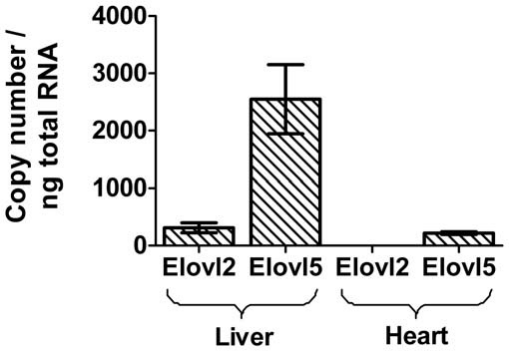
Comparison of the absolute number of Elovl2 and Elovl5 mRNA template copies / ng of total RNA in rat liver and heart. Elovl2 was detected in heart at <10 copies / ng of total RNA. All measurements were performed in duplicate and the results are the mean ± S.E.M. (n = 3).

The dose responses indicate that both elongase enzymes have similar affinities for EPA and therefore, both could produce DPA. However, expression levels *in vivo* are quite different with *Elovl5* being present at much higher levels than *Elovl2* in liver and heart (Fig. 4). The expression of both *Elovl5* and *Elovl2* was higher in liver compared to heart (Fig. 4).

### Δ6-Desaturase (Fads2)

#### Sequence analysis of *Fads2*

Amplification of the 1335 bp *Fads2* ORF and subsequent alignment with the GenBank sequence NM_031344 revealed one nucleotide difference. The *Fads2* sequence was deposited in GenBank as accession number HQ909027. The predicted Fads2 protein of 444 amino acids contained one residue difference compared to NP_112634. The residue change was not within the regions characteristic of fatty acyl desaturases; the conserved histidine boxes or cytochrome b_5_ domain containing the heme-binding motif.

#### Fads2 substrate specificities

**Figure 5:**
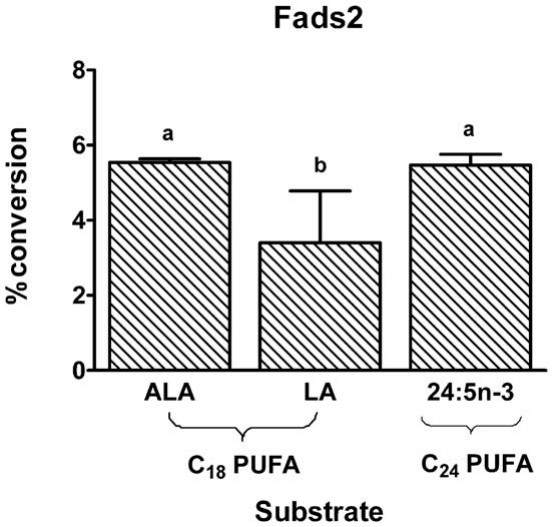
The rat Fads2 substrate specificity. Recombinant *S. cerevisiae* expressing Fads2 were grown in the presence of 100 µM of various PUFA substrates. Fatty acids were extracted from the recombinant *S. cerevisiae* and the amount of each fatty acid was expressed as a percentage of the total amount of all fatty acids. The proportion of substrate fatty acid converted to desaturation product was calculated as [product/(product + substrate)]×100. The product contained one more double bond than the substrate. The results are the means ± S.D. of triplicate incubations. Values with different symbols are significantly different from each other.

Recombinant *S. cerevisiae* cells expressing Fads2 were cultured in the presence of 100 µM of various C_18-24_ PUFA substrates to determine the enzymes substrate specificity. Fads2 demonstrated Δ6-desaturase activity with the expected n-3 and n-6 substrates, ALA and LA, and with 24:5n-3 (Fig. 5). Fads2 did not exhibit Δ5-desaturase activity with the substrates 20:4n-3 or 20:3n-6, or Δ8-desaturase activity with the substrates 20:3n-3 and 20:2n-6 (data not shown).

#### Competition between n-3 C_18_ and C_24_ substrates for Fads2

**Figure 6:**
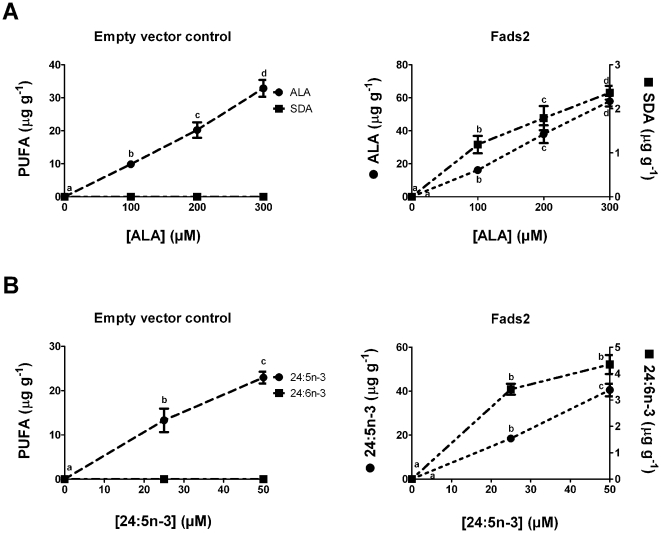
Examination of product/substrate relationships for desaturation by Fads2. Recombinant *S. cerevisiae* expressing empty pYES2 vector or Fads2 were grown in the presence of ALA (A) or 24:5n-3 (B). Fatty acids were extracted from the recombinant *S. cerevisiae* and the amount of each fatty acid was quantified in µg g^−1^ of *S. cerevisiae*. The results are the means ± S.D. of triplicate incubations. Values with different symbols are significantly different from each other.

**Figure 7:**
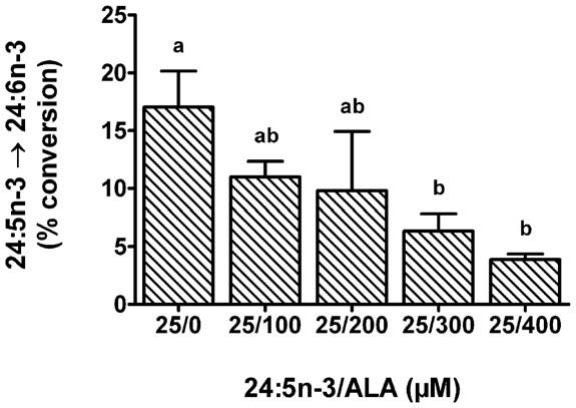
Competition between n-3 PUFA substrates for desaturation by Fads2. Recombinant *S. cerevisiae* expressing Fads2 were grown in the presence of 25 µM of 24:5n-3 and 0–400 µM ALA. Fatty acids were extracted from the recombinant *S. cerevisiae* and the amount of each fatty acid was expressed as a percentage of the total amount of all fatty acids. The proportion of substrate fatty acid converted to desaturation product was calculated as [product/(product + substrate)]×100. The results are the means ± S.D. of triplicate incubations. Values with different symbols are significantly different from each other.

ALA and 24:5n-3 were both Fads2 substrates with similar conversion efficiencies at a single concentration (Fig. 5). Therefore, the potential for competitive inhibition was examined at concentrations of ALA and 24:5n-3 that were in the linear response range (Fig. 6). The concentration of 24:5n-3 that was examined was much lower than the concentration of ALA. However, 24:5n-3 is likely to be lower than ALA *in vivo* because 24:5n-3 is usually not detectable in ALA dietary studies. Increasing concentrations of ALA over the range 0–400 µM dose-dependently inhibited the conversion of 24:5n-3 to 24:6n-3 (Fig. 7).

## Discussion

**Figure 8:**
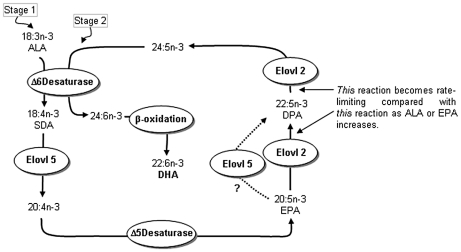
N-3 long-chain polyunsaturated fatty acid synthesis showing two-stages of Δ6-desaturase involvement and the individual elongase involvement. ? denotes that Elovl5 or Elovl2 can catalyse this reaction.

Our direct comparative study of the rat elongases showed that the substrates for Elovl5 are C_18_ and C_20_ PUFA, while for Elovl2 they are C_20_ and C_22_ PUFA (Fig. 8). This is generally concordant with specificities from separate studies of mouse Elovl2 [6], human Elovl2 [6], human Elovl5 [4] and rat Elovl5 [5], although there are some differences. The rELO1 (now Elovl5) examined by Inagaki *et al.* [5] had four residue differences from the Elovl5 examined in this study and it had markedly greater preferences for C_18_ over C_20_ substrates, both n-3 and n-6, which were not observed in our study. Also, compared with the rat Elovl2 in our study, the mouse Elovl2 appeared to convert EPA preferentially through to 24:5n-3 with less accumulation of DPA [6]. However, only a single concentration of EPA was used in the mouse study. We have shown that the proportion of EPA converted to 24:5n-3 by Elovl2 compared with that accumulating as DPA, diminishes with increasing EPA concentration. A possible explanation is that the second reaction, DPA to 24:5n-3, becomes saturated.

We also examined competitive substrate interactions. Elovl2 and Elovl5 each have at least two n-3 substrates, raising the potential for competitive substrate inhibition. Although SDA and EPA are both Elovl5 substrates, there was no evidence to suggest n-3 PUFA competition between substrates at the concentrations used in this study. Luthria and Sprecher [9] reported that neither EPA nor DPA inhibited SDA elongation in rat liver microsomes, although at the time they were unaware of which elongase enzyme was responsible. It is clear now that DPA does not inhibit SDA elongation because they are substrates for different enzymes, Elovl2 and Elovl5, respectively. However, we confirm that EPA does not appear to inhibit SDA elongation by Elovl5.

The sequential reactions of EPA→DPA→24:5n-3 performed by Elovl2 presented difficulties for examining possible competition between EPA and DPA (Fig. 8). Attempts were made to examine EPA/DPA competition, but results were uninterpretable due to DPA being both product and substrate with the same enzyme. Use of labelled EPA was considered, but was discounted due to similar concerns with interpretation of the results. However, the dose response of EPA with Elovl2 provided insights into the one enzyme dual reaction processes. With increasing EPA concentrations up to 400 µM, DPA increased proportionately whereas the subsequent product, 24:5n-3, reached a plateau at 200 µM EPA. This could reflect competition from EPA for the second reaction, DPA→24:5n-3. However, the simpler explanation is that the Elovl2 second reaction, DPA→24:5n-3, may be saturated at a lower substrate concentration than the first reaction, EPA→DPA. This explanation is supported by the demonstration of apparent saturation of the reaction DPA→24:5n-3 using DPA as the exogenous substrate. Possible saturation of the Elovl2 second reaction in the absence of saturation of the first reaction provides an additional explanation for the increase of DPA but not DHA after certain concentrations/intakes of ALA have been exceeded.

These conclusions are contingent in part on the expressed elongases being rate limiting for the process of elongation which requires the Elovl enzyme and three additional enzymes. The Elovl enzyme catalyses the initial condensation reaction while the enzymes required for the subsequent reduction-dehydration-reduction steps necessary for complete elongation are supplied by the yeast [10]. However, it has been shown that the condensation reaction is the rate-limiting step in elongation, even in a yeast heterologous expression system [10].

The rat Fads2 was active with the conventional Δ6-desaturase C_18_ and C_24_ PUFA substrates, but does not have Δ5- or Δ8-desaturase activity. This was unlike the baboon Fads2 which had Δ6- and Δ8-desaturase activity [11]. However, our findings with Fads2 are in agreement with previous studies that confirmed lack of Δ5-desaturase activity with the rat enzyme expressed in yeast or the mouse enzyme expressed in primary cultures of rat hepatocytes and in CHO cells, although these studies did not report activity values and did not examine C_24_ PUFA substrates [12], [13]. A later study indirectly examined the rat Δ6-desaturase activity towards the C_24_ substrates by expressing the enzyme in COS-7 cells [14]. The transfected cells were supplemented with DPA or 22:4n-6, relying on the endogenous cellular elongases to produce the Δ6-desaturase substrates 24:5n-3 and 24:4n-6 [14]. This study suggested that the rat Δ6-desaturase was more active towards ALA than 24:5n-3 although it is difficult to compare Δ6-desaturase activity when ALA was supplied exogenously but 24:5n-3 was formed endogenously.

Fads2 has been accepted as a control point in the production of DHA from ALA. Fads2 is an essential enzyme because DHA was not present or substantially reduced in Fads2 knockout mice [15], [16], [17]. The contribution of Fads2 in controlling the synthesis of DHA is two-fold. It is rate-limiting for conversion of ALA to EPA [1], [2], [3] and it also converts 24:5n-3, the product of Elovl2 activity, to 24:6n-3. Thus, there is the potential for competitive C_18_ and C_24_ n-3 substrate interactions and this could limit one or both reactions. Although it has been suggested there is competition between the C_18_ and C_24_ n-3 substrates for Fads2, it had not been investigated directly [7], [8]. The Fads2 dose response with ALA and 24:5n-3 was used to select a series of 24:5n-3/ALA ratios to examine competition. In ALA dietary studies with rats or humans where ALA is readily detectable in membrane phospholipids, 24:5n-3 is not detectable or barely detectable. Therefore, we examined ratios with ALA as the predominant fatty acid as we considered this would reflect *in vivo* ratios more closely compared with ratios in the other direction. Our study demonstrated that increasing concentrations of ALA can inhibit the conversion of 24:5n-3. This directly supports suggestions that the competition between ALA and 24:5n-3 may explain the decreasing DHA levels after certain intakes of ALA are exceeded [7], [8].

The present study indicates that Elovl2 is another potential control point, additional to that of Fads2. The scheme in Fig. 8 places the elongases according to their substrate selectivities. Although both Elovl2 and Elovl5 could catalyse EPA to DPA conversion, Elovl5 may be more important since our data show it is expressed at much higher levels than Elovl2 in rat liver and heart. However, neither EPA nor DPA elongation were diminished in the liver of *Elovl5^-/-^* mice, indicating that Elovl2 was responsible for EPA elongation in these knockout mice [18]. Nevertheless, Elovl2 was upregulated substantially in the *Elovl5^-/-^* mice compared with the wild type controls [18]. The serum of *Elovl2^-/-^* mice had elevated EPA and DPA levels [19]. However, Elovl5 may have been upregulated in the *Elovl2^-/-^* mice resulting in the accumulation of EPA and DPA, analogous to the upregulation of Elovl2 in *Elovl5^-/-^* mice, although this was not investigated [19]. Therefore, it remains uncertain if one or both elongases normally contribute to EPA elongation.

The scheme in Fig. 8 highlights the essentiality of Elovl2 activity for DHA formation. The finding that 24:5n-3 formation is possibly saturated at substrate concentrations not saturating for DPA formation, provides explanations for previous dietary findings. For example, tracer studies in healthy volunteers showed that increased EPA intake as a fish-based diet, increased the mass of EPA to DPA conversion, but decreased the mass of DPA to 24:5n-3 conversion [20]. The scheme proposed in Fig. 8 suggests these reported results could be due to increased EPA saturating the second Elovl2 reaction and competing with DPA for Elovl2 activity. The possible saturation of the second Elovl2 reaction provides an explanation for the limitations of both ALA and EPA as progenitors of DHA formation and provides an explanation for the accumulation of DPA when ALA or SDA or EPA is provided in the diet [1], [2], [3].

SDA enriched soybeans were granted Generally Recognised As Safe (GRAS) status by the US FDA in 2009 (http://www.soymega.com/en/Why-Soymega/). Bypassing the first Fads2 reaction has the potential to improve EPA and DPA tissue levels compared with standard soybean products. However, the present study suggests that Elovl2, both its presence and its activity, will be critical in understanding if DHA synthesis can be increased by dietary means.

Given the essentiality of Elovl2 for DHA synthesis, a functional Elovl2 may control the habitat and diet of a range of species such as fish, chickens, rats and humans. Freshwater fish and salmonids which consume herbivorous or omnivorous diets are generally considered to be capable of synthesizing DHA from ALA. Interestingly, zebrafish and Atlantic salmon are the only non-mammalian vertebrates with a functionally characterised Elovl2 [21], [22]. Searches of the ENSEMBL genomes suggest that the carnivorous fish species *Tetraodon nigroviridis* and *Gasterosteus aculeatus* and even omnivorous fish species *Takifugu rubripes* and *Oryzais latipes* do not contain Elovl2 homologs [22]. The lack of Elovl2 could impose an absolute block on their ability to synthesize DHA from ALA and accordingly Elovl2 may be a genetic marker for DHA synthetic capacity of a species.

## Materials and Methods

### Materials

All reagents were obtained from Invitrogen™ Australia Pty. Ltd. (Mount Waverley, Vic, Aust.) unless otherwise stated. The PUFA substrates and internal standard 17:0 were obtained from Nu-Chek Prep, Inc. (MN, USA), Cayman Chemical Company (Sapphire Bioscience, Australia) or Larodan Fine Chemicals (Malmö, Sweden).

### Cloning the *Elovl2*, *Elovl5* and *Fads2* cDNA

Total RNA was extracted from the livers of Dark Agouti rats with the RNeasy kit (Qiagen, Australia). RT-PCR amplification of the *Elovl2*, *Elovl5* and *Fads2* open reading frames (ORFs) was performed using the primers in Table 1 and OneStep RT-PCR Enzyme Mix (Qiagen). The primers were used to generate restriction enzyme sites flanking the ORFs. The resulting cDNAs and the expression vector pYES2 were restriction enzyme treated and ligated using T4 DNA ligase (1.5 Weiss units) (Promega, WI, USA). Transformation of the resulting constructs, pYES2-*Elovl2*, pYES2-*Elovl5* and pYES2-*Fads2* into MAX Efficiency® DH5α™ Competent *E. coli* cells was performed using heat-shock. Putative transformants were selected using 100 µg mL^-1^ ampicillin and PCR screening. Recombinant plasmids were purified and the presence of the gene insert in the correct orientation was confirmed via sequencing at the Institute of Medical and Veterinary Science (Adelaide, Australia).

**Table 1:** Primers used for amplifying the *Elovl2*, *Elovl5* and *Fads2* ORFs, including the GenBank accession number of the sequence used for primer design.

Gene	Primer	Sequence 5′→3′	Accession number
Elovl2	Elovl2-F	GCG*GAATTC*TTGGACAACATGTTTGGACCA	NM_001109118
	Elovl2-R	GACT*GCGGCCGC*CGCTTCACCTCATTGCACCTTCTTG	
Elovl5	Elovl5-F	CCC*GGATCC*AAAATGGAACATTTCGATGCG	NM_134382
	Elovl5-R	CCG*CTCGAG* TCAATCCTTCCGGCTGCTTCC	
Fads2	Fads2-F	GCG*GAATTC*ACAGGCAGCATGGGGAAGGGA	NM_031344
	Fads2-R	GACT*GCGGCCGC*TCTGCTGCTTCATTTGTGGAG	

Restriction enzyme sites are indicated by italics and the start and stop codons are underlined.

### Heterologous expression of the Elovl2, Elovl5 and Fads2 ORFs in Saccharomyces cerevisiae

The pYES2-*Elovl2*, pYES2-*Elovl5* and pYES2-*Fads2* constructs were used to transform *Saccharomyces cerevisiae* strain INVSc1 for the production of recombinant protein, using the *S. c.* EasyComp™ Transformation Kit. Successfully transformed yeast cells were selected on uracil dropout medium. Recombinant yeast cells were maintained in synthetic minimal defined medium for yeast without uracil containing 2% glucose. Upon fatty acid supplementation, glucose was replaced with 2% galactose for induction of gene expression.

Recombinant yeast expressing Elovl2 or Elovl5 were supplemented with one or more of the following PUFA substrates: 18:4n-3 (SDA), 18:3n-6 (γ-linolenic acid; GLA), 20:5n-3 (EPA), 20:4n-6 (arachidonic acid; AA), 22:5n-3 (DPA) or 22:4n-6.

Recombinant yeast expressing Fads2 were supplemented with one or more of the following putative Δ6-desaturase substrates: 18:3n-3 (ALA), 18:2n-6 (linoleic acid; LA) or 24:5n-3; Δ5-desaturase substrates: 20:4n-3 or 20:3n-6; Δ8-desaturase substrates: 20:3n-3 or 20:2n-6.

PUFA substrates were prepared in ethanol. The volume of ethanol in each incubation was kept constant. Cells were harvested for analysis after 24 h of fatty acid supplementation at 30°C. The cells were pelleted by centrifugation at 1500 *g* for 5 min and the cell pellets were washed with 0.85% saline. The wet weights of the cell pellets were recorded. Data are expressed as the means ± SD of triplicate incubations for each treatment.

### *Elovl2* and *Elovl5* expression levels

Dark Agouti rats consumed a fat free AIN-93G rodent diet (Glen Forrest Stockfeeders, Glen Forrest, Western Australia) blended with flaxseed oil to obtain 1%en ALA and 2.1%en LA, to a level of 5% (w/w) fat. The experiment was approved by the Animal Ethics Committee of the Institute of Medical and Veterinary Science (125/06) and was performed in accordance with the National Health and Medical Research Council Australian Code of Practice for the Care and Use of Animals for Scientific Purposes. Total RNA was extracted from the liver or heart of the rats with the RNeasy kit (Qiagen). The quantity and quality of RNA was determined by measuring the absorbance at 260 and 280 nm (NanoDrop Technologies Inc., DE, USA) and agarose gel electrophoresis. Total RNA (0.5 µg) was reverse transcribed into cDNA using the QuantiTect® Reverse Transcription kit (Qiagen). Quantitative reverse transcription-polymerase chain reaction (qRT-PCR) was performed using a Rotor-Gene 3000 (Corbett Research) with the QuantiFast™ SYBR® Green PCR kit (Qiagen). Each reaction contained 10 ng of cDNA, 5 µl QuantiFast™ SYBR® Green PCR master mix and 1 µl of QuantiTect® Primer Assay (Qiagen), in a total volume of 10 µl. The QuantiTect® Primer Assays used were Elovl2 (Rn_Elovl2_2_SG, QT01683899) and Elovl5 (Rn_Elovl5_1_SG, QT00178059). Elovl2 or Elovl5 PCR products were used to make 10-fold serial dilutions from 10^6^ copies down to 10 copies. The PCR product standard curve equations were used to calculate the absolute copy number of Elovl2 and Elovl5 in rat liver and heart. The cycling conditions were as follows: PCR initial activation step at 95°C for 5 min, 2 step cycling with 35 cycles of denaturation at 95°C for 10 s and annealing/extension at 60°C for 30 s. Each amplification was performed in duplicate.

### Fatty acid analysis

Total lipid was extracted from yeast cells and analysed by gas chromatography as previously described [23]. The identities of 20:4n-3 and 22:4n-3 were previously confirmed by gas chromatography–mass spectrometry [23]. The internal free fatty acid standard 17:0 was used to quantify the amount of each endogenous fatty acid. The proportion of substrate fatty acid converted to fatty acid product(s) was calculated as [product(s)/(product(s) + substrate)]×100.

### Statistical analysis

One-way ANOVA with Tukey's post-hoc test was performed using Graphpad Prism version 5.03 for Windows (Graphpad Software, San Diego, CA, USA). Statistical significance was set at *P*<0.05.
